# Fibrin-Enhanced Canonical Wnt Signaling Directs Plasminogen Expression in Cementoblasts

**DOI:** 10.3390/ijms18112380

**Published:** 2017-11-09

**Authors:** Saeed Ur Rahman, Chan Ho Park, Jeong-Hwa Baek, Hyun-Mo Ryoo, Kyung Mi Woo

**Affiliations:** 1 Department of Molecular Genetics, Dental Research Institute and BK21 Program, School of Dentistry, Seoul National University, Seoul 08826, Korea; saeedbio80@gmail.com (S.U.R.); perioengineer@snu.ac.kr (C.H.P.); baekjh@snu.ac.kr (J.-H.B.); hmryoo@snu.ac.kr (H.-M.R.); 2 Department of Pharmacology & Dental Therapeutics, School of Dentistry, Seoul National University, Seoul 08826, Korea

**Keywords:** fibrin, cementoblast, plasminogen, Wnt3a, β-catenin

## Abstract

Cementum is a mineralized layer on the tooth’s root surface and facilitates the biomechanical anchoring of fibrous connective tissues as a part of tooth-supportive complexes. Previously, we observed that OCCM30 cementoblasts cultured on fibrin matrices underwent apoptosis due to fibrin degradation through the expression of proteases. Here, we demonstrated that OCCM30 on fibrin matrices (OCCM30-fibrin) enhanced canonical Wnt signaling, which directed to plasminogen expression. The OCCM30-fibrin showed higher levels of Wnt3a expression, nuclear translocation of β-catenin, and T-cell factor (TCF) optimal motif (TOP) reporter activity than the cells on tissue culture dishes (OCCM30-TCD), indicating that the OCCM30-fibrin enhanced canonical Wnt/β-catenin signaling. Also, OCCM30-fibrin expressed biomineralization-associated markers at higher levels than OCCM30-TCD, of which levels were further increased with LiCl, a Wnt signaling activator. The OCCM30 cementoblasts simultaneously showed that high levels of plasminogen, a critical component of fibrinolysis, were expressed in the OCCM30-fibrin. Activation of canonical Wnt signaling with LiCl treatment or with forced lymphoid enhancer factor 1 (LEF1)-expression increased the expression of plasminogen. On the contrary, the inhibition of canonical Wnt signaling with siRNAs against Wnt3a or β-catenin abrogated fibrin-enhanced plasminogen expression. Furthermore, there are three conserved putative response elements for the LEF1/β-catenin complex in the plasminogen proximal promoter regions (−900 to +54). Site-directed mutations and chromatin immunoprecipitation indicated that canonical Wnt signaling directed plasminogen expression. Taken together, this study suggests that fibrin-based materials can modulate functional periodontal formations in controlling cementoblast differentiation and fibrin degradation.

## 1. Introduction

As the paradigm shifts from tissue replacement by prosthetics to tissue regeneration in orthopedic or dental research, engineered microenvironments and localized delivery systems of biological factors have rapidly been developed for preclinical or clinical wound healing [1,2,3]. For a few decades, natural or synthetic biopolymers have been fabricated for biomimetic extracellular matrices (ECMs) with biocompatibility and biodegradability [4]. Natural polymers with various chemically-conjugated biologics in materials facilitate the enhancement of cell–material interactions [5,6] and the improvement of various bioactivities for tissue morphogenesis [7,8]. Among the natural polymers, fibrin matrices (1) exhibit controllable biodegradation rates with precursor concentrations of fibrinogen and thrombin [9]; (2) are easily fabricated to create customized spatial constructs by enzymatic polymerization of plasma protein (fibrinogen) with activated proteolytic enzyme (thrombin) [10,11]; and (3) have minimal inflammation for a normal wound healing process [12] for preclinical or clinical scenarios. Due to their significant characteristics, fibrin gels have been modified to improve their properties and have been widely used to deliver quantified cells for tissue regeneration, such as bone [11], cartilage [13], cardiovascular tissues [14], or nerve tissues [15]. In particular, biodegradability is a critical property to provide spatiotemporal microenvironments for cell migration, proliferation or differentiation over time and ultimately enable tissue formation and maturation [16,17,18]. The biodegradation process of fibrin material, fibrinolysis or the fibrinolytic system is significantly mediated by plasmin [19], which is formed from plasminogen cleavage by plasminogen activators (tissue-type plasminogen activator; t-pA and urokinase-type plasminogen activator; u-pA) Inhibition of fibrinolysis leads to tissue regeneration or tissue remodeling using plasminogen activator inhibitor-1 (PAI-1) or α2-antiplasmin (α2Ap) that commonly neutralizes plasminogen activators and blocks the interactions between fibrin structures and the lysine-binding domain of plasminogen, which can have an important role in fibrinolysis [20]. Therefore, the degradability or sustainability of fibrin matrices can be controlled by biologics and their biochemical contributions which are mediated by cell–fibrin matrix interactions such as with fibrin, plasminogen, and plasminogen activators [21,22].

For physiological restorations in a functional periodontal complex, interfacial tissues are significantly required to structurally integrate individual tissues and to form hierarchical constructs, like the bone–periodontal ligament (PDL)–cementum–tooth surface [23]. In particular, the cementum has a pivotal role in systematically serving tooth-supportive functions [24], because the PDL bundles anchored by the cementum on the tooth’s root surface can pertinently respond to masticatory/occlusal loadings and movements for tooth-supportive functions [23]. The cementum is a mineralized tissue layer on the tooth’s root surface with a 250–300 μm thickness and cementogenesis provides functional integration by inserting Sharpey’s fibers, which are the terminal ends of the predominantly type III collagen bundles in the PDL [25,26].

The Wnt signaling pathway has been known to affect tooth morphogenesis [27,28,29,30] and compartmentalized functional tissue integrations in periodontal complexes are exclusively promoted at the tooth-developmental stage by the canonical Wnt signaling pathway [27,28,29,30,31], which is one of the three typical Wnt-signaling categories, including the noncanonical Wnt-planar cell polarity pathway and the non-canonical Wnt/calcium pathway [19]. Briefly, the canonical Wnt signaling pathway results in nuclear translocation of β-catenin and then the complex of β-catenin and T-cell factor/lymphoid enhancer factor (T-cell factor (TCF)/LEF) can be formed to transcriptionally activate mineralized tissue formations [19,31,32]. Nemoto et al. demonstrated that the canonical Wnt signaling pathway can promote cementoblast proliferation in vitro [33] and the Wnt/β-catenin signaling pathway has recently been reported to activate the cementogenic differentiation of PDL cells and to induce cementogenesis [31,34]. Including the Wnt signaling pathway, various upregulating factors for bio-mineralization can significantly influence cementoblast differentiation [35,36] like Runx-related gene 2 (Runx2), alkaline phosphatase (ALP), bone sialoprotein (BSP), or osteocalcin (OCN) and cementogenesis is consequently promoted on the tooth’s root surface with similar biochemical compositions as other mineralized tissues [37].

Previously, we investigated the differentiation of MC3T3-E1 pre-osteoblasts by natural biopolymeric materials like fibrin and collagen [1,38,39]. In particular, fibrin significantly promoted osteoblast differentiation by the large capacity of fibronectin binding which can regulate pre-osteoblast activities [39,40] and by the optimal ratio of thrombin, which can polymerize fibrinogen to fibrin [38]. However, cementogenesis using biochemical or engineered approaches still remains a challenge because of the limitation to form a micron-scaled mineralized layer with morphological compartmentalization to the adjacent fibrous connective tissues, the PDL. We hypothesize that OCCM30 cells on a fibrin gel can have cementoblast differentiation by activating the canonical Wnt signaling pathway; however, Wnt signaling simultaneously affects plasminogen expression to induce matrix degradation. In this study, cementoblast–fibrin interactions were investigated for fibrin-enhanced canonical Wnt signaling leading to plasminogen expression.

## 2. Results

### 2.1. OCCM30 Cultured on Fibrin Enhances Canonical Wnt Signaling

**Figure 1:**
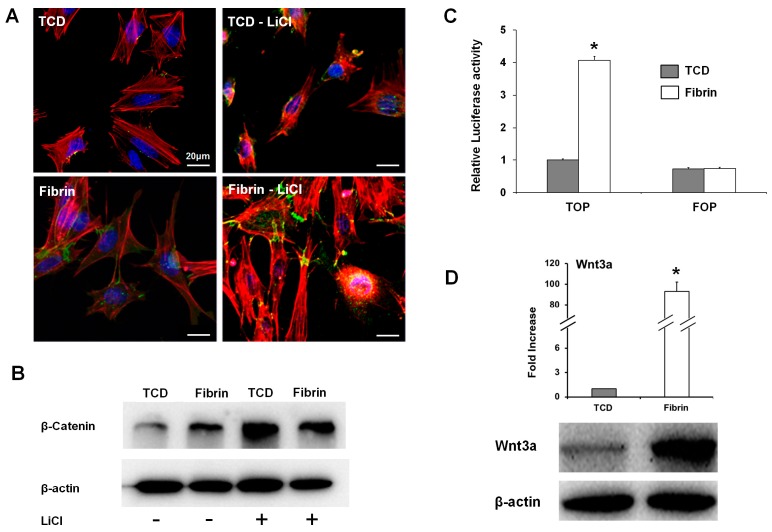
OCCM30 cultured on fibrin enhances canonical Wnt signaling. OCCM30 cells were cultured on fibrin and tissue culture dish (TCD) in differentiation media over a three-day period with/without LiCl. Fibrin induced the translocation of β-catenin from the cytoplasm to the nucleus in cementoblasts (green-fluorescence represented β-catenin): OCCM-30 cells were cultured on fibrin and TCD for three days, and the nuclear translocation of β-catenin detected by immunofluorescence showed that the protein translocated to the nucleus was increased in the cells cultured on fibrin (**A**). β-catenin expression was also determined by immunoblot analysis and it was highly expressed rather than TCD (**B**). Cementoblast cells were transfected with Top flash (wild-type promoter) or Fop flash (mutant promoter) reporter plasmids and cultured in differentiation media for the last 24 h, and luciferase activities were measured in cell lysates and normalized to a *Renilla* transfection control (**C**). Fibrin exhibited a higher expression of Wnt3a and induced canonical Wnt signaling which was determined by quantitative real-time polymerase chain reaction (PCR) and western blot analysis (**D**). Each experiment was repeated two times independently. * Significantly different (*p* < 0.05).

It was investigated whether fibrin enhanced canonical Wnt signaling by comparing OCCM30 cells cultured on fibrin (OCCM30-fibrin) with cells cultured on TCD (OCCM30-TCD). Expression of Wnt3a, which can trigger the canonical Wnt signaling pathway, was checked in cells cultured for three days in a differentiation medium. The immunofluorescence microscope images showed that β-catenin was more intensely stained in the OCCM30-fibrin and that its nuclear location was increased (Figure 1A). As a positive control for the activation of the canonical Wnt signaling pathway, LiCl treatment was used, which inhibits glycogen synthase kinase-3β activity, increases β-catenin stabilization, and induces nuclear translocation to interact with the TCF/LEF family members to form complexes [32,41,42]. After the OCCM30 cells were treated with LiCl for 24 h, the nuclear accumulation of β-catenin increased in both the cells on fibrin and TCD; however, it was clearly evident in the OCCM30-fibrin (Figure 1A and Figure S1). Western blot analysis for β-catenin also indicated that the levels of β-catenin were higher in the OCCM30-fibrin (Figure 1B). In the TOP flash luciferase reporter assay, it was shown that the transcriptional activity of the β-catenin complexes was higher in the OCCM30-fibrin, while far from optimal motif (FOP) flash was used as a negative control and showed no differences in the transcriptional activity between both the fibrin and TCD groups (Figure 1C). The OCCM30-fibrin was expressed at much higher levels of Wnt3a transcripts and the protein was also expressed at much higher levels than that of the OCCM30-TCD (Figure 1D). These results indicate that canonical Wnt signaling was activated and enhanced in the OCCM30-fibrin.

**Figure 2:**
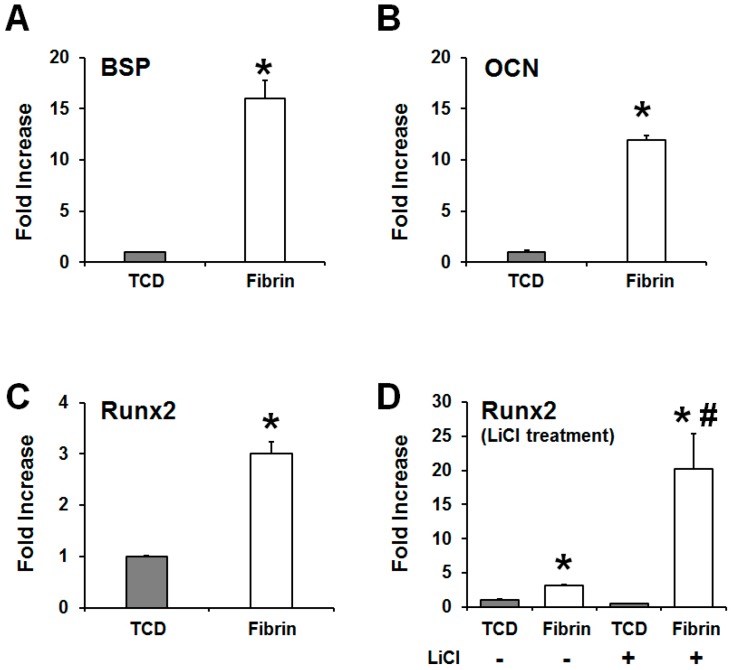
Activation of canonical Wnt signaling in OCCM30 on fibrin and the promotion of the expression of biomineralization-associated molecules. OCCM-30 cells were cultured on TCD (OCCM30-TCD) and fibrin matrices (OCCM30-fibrin) to accelerate cementoblast differentiation for three days. Using quantitative real time PCR, biomineralization-associated molecules had higher expression levels in the fibrin matrices; alkaline phosphatase (ALP; (**A**)), Osteocalcin (OCN; (**B**)), runt-related gene 2 (Runx2; **C**), and Runx2 with/without the LiCl treatment (**D**). All biomineralization-associated factors can be highly expressed in OCCM30-fibrin with statistically significant differences (**A**–**C**). Particularly, despite the intentional activation of the canonical Wnt signaling by the LiCl treatment, LiCl-treated OCCM30-TCD showed lower Runx2-expression level than OCCM30-fibrin (without the LiCl treatment; (**D**)). They were normalized to mRNA levels of glyceraldehyde-3-phosphate dehydrogenase (GAPDH) and indicated by fold increase. Each experiment was repeated two times independently. * Significantly different from the OCCM30-TCD groups (*p* < 0.05), ^#^ significantly different from LiCl non-treated group (*p* < 0.05).

In addition, quantitative real-time PCR was used to analyze the transcript levels of cementoblast/osteoblast differentiation markers in the OCCM30-fibrin and OCCM30-TCD. The results show that the OCCM30-fibrin had statistically higher levels of the molecular expression of BSP, OCN, and Runx2, which were identified for biomineralization or mineralized tissue formations (Figure 2A–C). Regardless of the LiCl treatments as the positive control, OCCM30-TCD showed statistically low expression levels of Runx2, but OCCM30-fibrin had statistically significant increases of Runx2 expressions (Figure 2D). Therefore, OCCM30-fibrin can more strongly activate cementogenic differentiation and promote the expression of biomineralization-associated molecules. 

### 2.2. Plasminogen Expression in OCCM30-Fibrin for the Fibrin Degradation

**Figure 3:**
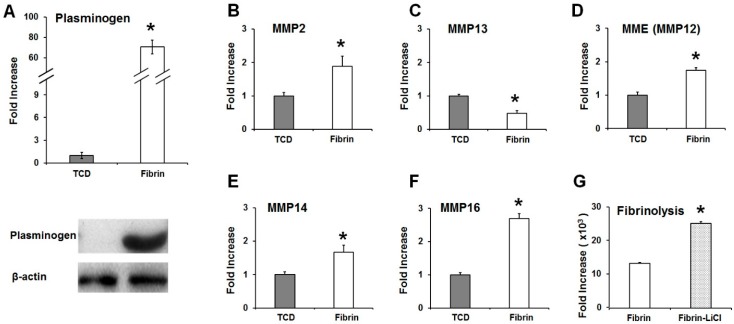
The OCCM30 on fibrin-enhanced expression of plasminogen. OCCM30-fibrin had a significantly higher expression level of plasminogen than that of the OCCM30-TCD (**A**) as well as fibrinolytic elements like matrix metalloproteinases (MMPs) (**B**–**F**). Moreover, LiCl treatment for the activation of canonical Wnt signaling as a positive control can lead to a fibrinolytic process in the OCCM30-fibrin like OCCM30-fibrin without the LiCl treatment even though there was a statistically significant difference (**G**). Each experiment was repeated two times independently. * Significantly different (*p* < 0.05).

Real-time qPCRs and western blots showed that the expression of plasminogen was significantly higher in the OCCM30-fibrin than in the OCCM30-TCD (Figure 3A). Plasminogen contributes to the degradation of fibrin matrices by binding to plasminogen activators like t-PA and u-PA and converting to plasmin. Expressions of matrix metalloproteinases (MMPs) were quantitatively analyzed in both the OCCM30-fibrin and OCCM30-TCD (Figure 3B–F). Statistical quantifications showed that MMP2 (gelatinase), MMP14 (membrane-type matrix metalloproteinase 1) membrane-type matrix metalloproteinase, MMP16 (type I transmembrane in furin-activatable MMPs), and MME (macrophage metalloelastase or MMP12) [43] had higher levels in the OCCM30-fibrin rather than in the OCCM30-TCD. In the case of MMP13 (collagenase in archetypal MMPs) [43], a statistically higher level was observed in the OCCM30-TCD instead of the OCCM30-fibrin (Figure 2C). Interestingly, the activation of the canonical Wnt signaling pathway by the LiCl treatment also increased the fibrinolysis in the OCCM30-fibrin even though it had the statistical difference (Figure 3G). Based on the results, the canonical Wnt signaling was significantly activated in the OCCM30-fibrin and the plasminogen expressions were highly increased with biodegradation enzymatic factors. Therefore, the canonical Wnt signaling can influence the plasminogen expression and the mechanism consequently could contribute biodegradation factors and fibrinolysis (Figure 3).

### 2.3. Fibrin-Enhanced Canonical Wnt Signaling in OCCM30 Directs Plasminogen Expression

**Figure 4:**
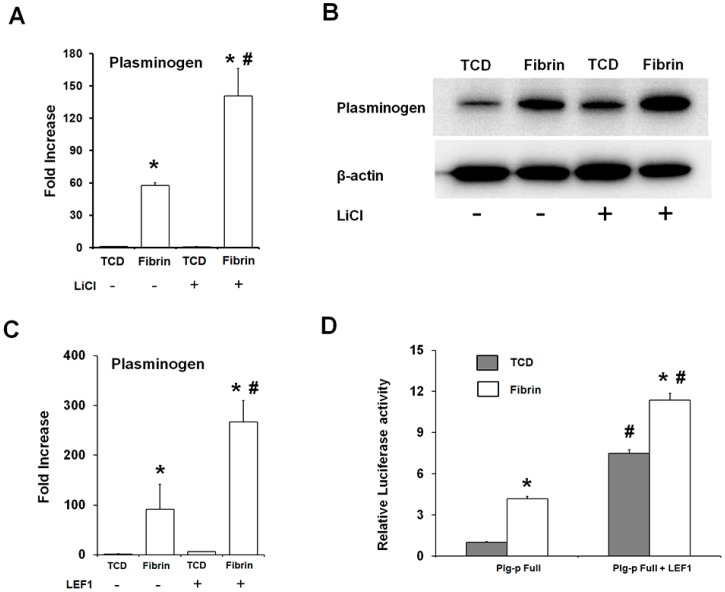
Activation of canonical Wnt signaling in the OCCM30-fibrin increases the expression of plasminogen. OCCM30 were cultured for three days in a differentiation medium. LiCl increased the plasminogen expression of the transcripts (**A**) and protein (**B**) in OCCM30-fibrin. Over-expression of LEF1 increased the expression of plasminogen (**C**) and the transcriptional reporter activity of the plasminogen promoter (**D**). Firefly luciferase activities were normalized with the *Renilla* luciferase activities. Each experiment was repeated two times independently. * Significantly different from the OCCM30-TCD groups (*p* < 0.05), ^#^ significantly different from LiCl or LEF1 non-treated group (*p* < 0.05).

The relationship between the fibrin-enhanced canonical Wnt signaling and the expression of plasminogen was determined. The activation of canonical Wnt signaling with LiCl treatment led to a further increase in the transcript and protein levels for plasminogen expression in the OCCM30-fibrin (Figure 4A,B). In the canonical Wnt signaling pathway, the β-catenin in the nucleus forms a complex with LEF1 to activate transcription of the target gene [19]. The forced expression of LEF1 significantly increased plasminogen expression, especially in the OCCM30-fibin (Figure 4C). In a plasminogen promoter (−900 b)-luciferase reporter construct, LEF1 overexpression led to enhanced luciferase activity in the OCCM30-fibrin (Figure 4D).

**Figure 5:**
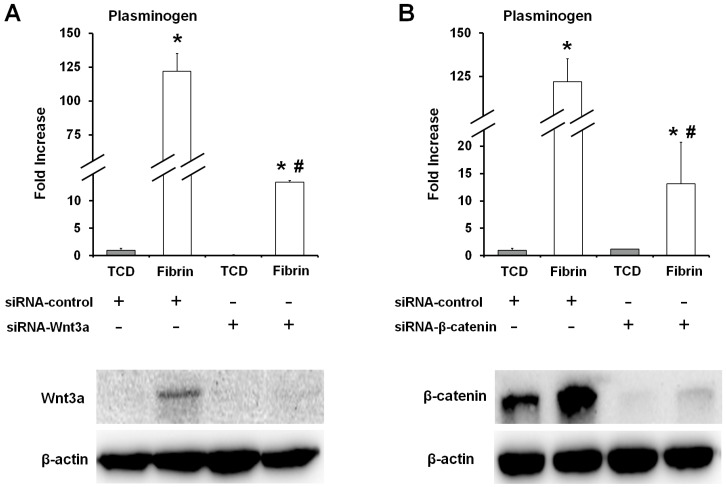
Knockdown of Wnt3a or β-catenin abrogated the fibrin-enhanced plasminogen expression. OCCM-30 cells were transfected with siRNAs against Wnt3a (**A**) and β-catenin (**B**). After 48 h of transfection, the protein levels of Wnt3a and β-catenin were detected by immunoblotting and plasminogen mRNA expression was determined by quantitative real-time PCR normalized to GAPDH. Wnt3a was specifically expressed in OCCM30-fibrin rather than other groups (**A**) but β-catenin expression was determined in OCCM30-TCD and OCCM30-fibrin (**B**). The knockdown models of Wnt3a and β-catenin had no expressions levels. Each experiment was repeated two times independently. * Significantly different from the OCCM30-TCD groups (*p* < 0.05), # significantly different from the si-RNA control groups (*p* < 0.05).

In order to determine the correlation between the canonical Wnt signaling pathway and the plasminogen gene expression in the OCCM30-fibrin, plasminogen expression was analyzed after knockdown of Wnt3a and β-catenin with siRNAs. From the results, the knockdown of Wnt3a and β-catenin dramatically reduced the expression of plasminogen in the OCCM30-fibrin (Figure 5). Based on the higher expression of Wnt3a in OCCM30-fibrin than other groups (Figure 5A), the fibrin matrices can specifically contribute Wnt/β-catenin signaling activation even though β-catenin was expressed in both OCCM30-TCD and OCCM30-fibrin (Figure 5B).

**Figure 6:**
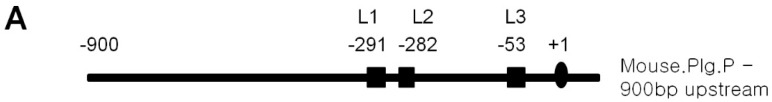

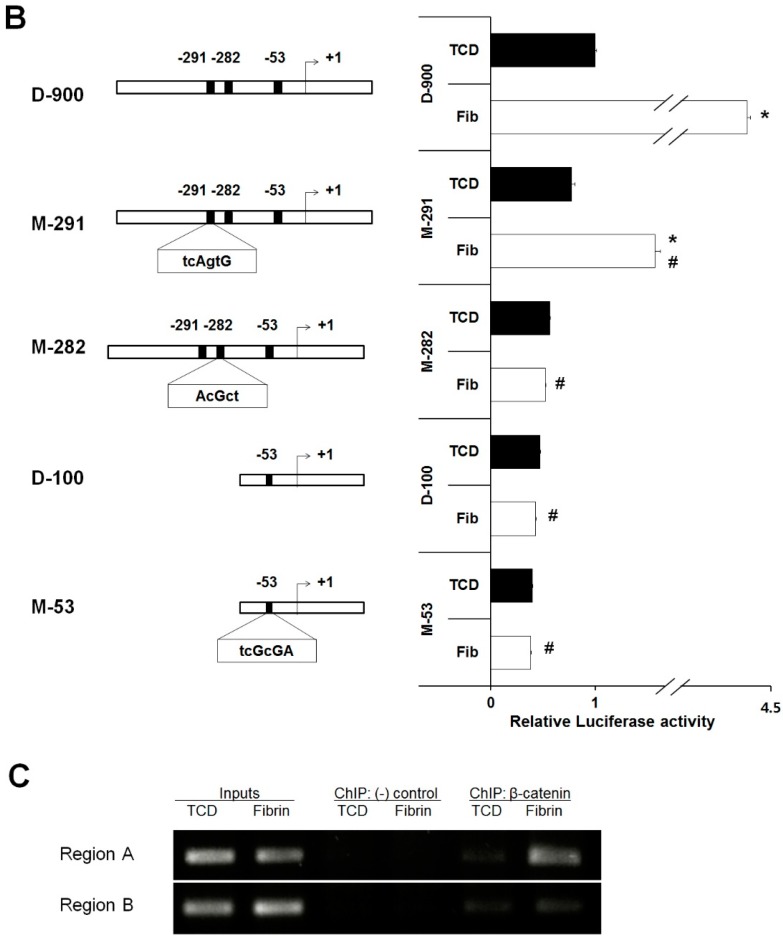
Plasminogen is a direct target of the canonical Wnt signaling in the OCCM30-fibrin. There are three putative response elements for the nuclear mediator of Wnt signaling LEF1 in the mouse plasminogen proximal promoter regions (−900 to +54). The binding positions are shown (**A**). Transient transfection was performed with luciferase reporter constructs containing the plasminogen promoter regions wild type (D-900 and a deletion construct D-100), mutants (M-291, M-282, and M-53), and an empty basic plasmid pGL-3 as a control. Firefly luciferase activities were quantified and normalized with the *Renilla* luciferase activities (**B**). Chromatin immunoprecipitation assay was performed to confirm the binding of β-Catenin to the LEF1 putative binding sites (Region A: L1 & L2; Region C: L3) on the plasminogen promoter (**C**). Each experiment was repeated two times independently. * Significantly different from the OCCM30-TCD groups (*p* < 0.05), ^#^ significantly different form from D-900 in the OCCM30-fibrin group (*p* < 0.05).

To address whether the canonical Wnt signaling in the OCCM30-fibrin directly targets plasminogen expression, the mouse plasminogen gene proximal promoter was analyzed by TESS, and putative LEF1 binding sites were found between −900 and the putative transcription start site (+1) denoted as follows: L1, −291 to −286, L2, −282 to −277, and L3, −53 to −48 respectively (Figure 6A). Site-directed mutants (M-291, M-282, and M-53) as well as the full length (D-900) and a deletion of the plasminogen promoter at the 5´end were transiently transfected into OCCM30 cultured on fibrin and TCD. The luciferase reporter assay was performed for these plasminogen promoter constructs, and it was found that the OCCM30-fibrin exhibited higher luciferase activity for the full-length construct (D-900). The site-directed mutants, M-291 and M-282, lost the reporter activities. Especially, M-282 did not show any differences in the luciferase activities between the OCCM30-fibrin and the OCCM30-TCD (Figure 6B). A deletion construct (D-100) and a site-directed mutant (M-53) showed only basal levels of luciferase activities. A ChIP assay was performed to determine whether the β-catenin/TCF/LEF complexes directly interact at the putative binding sites. Chromatin was prepared from the OCCM30 cells and immunoprecipitated with an antibody specific for β-catenin. After the immunoprecipitation, DNA was purified and used as a template for amplification using specific primers for PCR corresponding to the regions of the mouse plasminogen promoter. We checked two regions, A and B, where the β-catenin forms a complex to bind to the TCF/LEF binding sequences, L1/L2 and L3, respectively. The binding was increased at region A in the cells on fibrin compared to the cells on TCD (Figure 6C). In contrast, there was no difference observed in the DNA-protein complex at region B of the plasminogen promoter among both groups. These results suggest that the β-catenin can interact with the TCF/LEF1 motifs to form a complex at region A of the mouse plasminogen promoter, which could increase the plasminogen transcription in the OCCM30-fibrin compared to the OCCM30-TCD. Taken together, these results show that canonical Wnt signaling in the OCCM30-fibrin directed the expression of plasminogen and that the regions of L1 and L2 could be the binding sites.

## 3. Discussion

Fibrin is a natural biopolymer material from critical blood-clotting components and formed by polymerization upon the thrombin-mediated cleavage of fibrinogen. This biomedical material is generally used to induce tissue formation as injectable biodegradable scaffolds or to deliver cell-encapsulated particles to target wound defects [44,45]. In addition to delivery systems, fibrin materials have been investigated and developed for various applications like bio-inkjet printing [46] or the self-assembly technique [47]. Particularly, fibrin can be easily tuned with various biocompatible components to improve its mechanical/chemical properties and to fabricate biodegradability controls for spatiotemporal tissue formations compared with collagen matrices [9,10,11]. Recently, we reported that the control of fibrinolysis can promote cementogenic differentiation in vitro and cementum formation on the tooth’s root surface in a canine model in vivo [1]. Here, we demonstrated that fibrin matrices enhanced canonical Wnt signaling with increased expression of plasminogen and also bio-mineralization-associated molecules, simultaneously. 

With the modification to slow fibrinolysis using aminocaproic acid [38,48] in the OCCM30 cell cultures, OCCM30-fibrin showed significantly higher expression of osteogenic/cementogenic differentiation molecules (BSP, OCN, and Runx2) than OCCM30-TCD (Figure 2A–C). Based on the results to promote the cementogenic differentiation, we examined a potential mechanism, the canonical Wnt signaling pathway, which is known to significantly contribute to osteoblast differentiation and to promote osteogenesis and periodontal tissue regeneration [19,49,50,51,52]. Our results clearly showed that OCCM30-fibrin enhanced Wnt3a/β-catenin signaling (Figure 1). Runx2, which is essential for osteoblastic differentiation in skeletal tissue formation, was highly expressed and further enhanced with LiCl treatment in the OCCM30-fibrin group (Figure 2C,D). These results indicated that the fibrin matrices activated the canonical Wnt signaling in OCCM30 cells and this activation contributed to osteogenic/cementogenic differentiations (Figure 2D). In addition to the analyses of biomineralization molecules and different enzymes for matrix degradations (Figure 3), plasminogen, a component responsible for the fibrinolytic process was highly expressed in OCCM30-fibrin, and its expression further increased with LiCl treatment (Figure 3 and Figure 4A,B). To rule out the possibility that the plasminogen in bovine serum used for culturing the cells would interfere with the analyses of plasminogen expression, we validated that the anti-plasminogen antibody used in this study did not detect the bovine plasminogen (data not shown). The forced expression of LEF1 also led to increases in plasminogen expression (Figure 4C) and plasminogen promoter activity (Figure 4D). For systematic correlations between the canonical Wnt signaling and plasminogen expression in OCCM30-fibrin, the reduction of Wnt signaling by the knockdowns of Wnt3a and β-catenin resulted in retractions of plasminogen expression with statistical differences (Figure 5). That is, the canonical Wnt signaling in OCCM30-fibrin can be the critically interactive factor in the plasminogen expression. Furthermore, we found that the nuclear β-catenin complex might directly enhance the transcription of plasminogen, confirmed by site-directed mutagenesis and ChIP assay (Figure 6). 

Cementogenic differentiation in OCCM30-fibrin can be simultaneously affected by fibrin degradation, which can lead to a loss of cell attachment and ultimately apoptosis. Therefore, natural fibrin matrices should be optimally modified to slow the rate of fibrin biodegradation, to enhance cementoblast differentiation, and to promote mineralization. We reported phenomenological results about the interactions of a fibrin biomaterial and OCCM30 cells for fibrinolysis and the promotion of cementogenesis in vitro and in vivo [1]. This study showed that canonical Wnt signaling can be the critical concern to prove the cell–material interactions, which simultaneously showed biodegradation and cementogenic differentiation. It is implicated that the challenging cementogenesis in vivo can be controlled and optimized to form a functioning interfacial tissue between mineralized and fibrous connective tissues. 

## 4. Materials and Methods

### 4.1. Reagents and Antibodies

Glycerol-2-phosphate, l-ascorbic acid, lithium chloride (LiCl), plasminogen-free fibrinogen from bovine plasma, thrombin, 6-aminocaproic acid, and Cholroform:Isoamyl alcohol 24:1 was purchased from Sigma-Aldrich Co. (St. Louis, MO, USA). Calcium chloride was purchased from Georgia Chemical Inc. (Suwanee, GA, USA). The WEST-ZOL™ Western Blotting System was purchased from iNtRON Biotechnology (Kyungki, Korea). RNAiso Plus reagents, PrimeScripts™ RT reagent kit, and SYBR^®^ Premix Ex Taq™ were purchased from Takara Bio Inc (Kyoto, Japan). VECTASHIELD^®^ Mounting Medium with 4′,6-diamidino-2-phenylindole (DAPI) was purchased from Vector Laboratories, Inc. (Burlingame, CA, USA). The Dual-Glo^®^ Luciferase assay kit and pRL *Renilla* Luciferase Control Reporter Vectors were purchased from Promega Co. (Madison, WI, USA). Genefectine™ reagent was purchased from Genetrone Biotech (Jeeonbuk, Korea). siRNA against Wnt3a and β-catenin of siGENOME SMARTpool, siGENOME Non-Targeting siRNA Pools #1, and ON-TARGET*plus* siRNA reagents were purchased from Dharmacon Inc. (Lafayette, CO, USA). The Protein G Agarose/Salmon Sperm DNA kit was purchased from Millipore (Kankakee, IL, USA). cOmplete™, an ethylenediaminetetraacetic acid (EDTA)-free protease inhibitor cocktail tablet was purchased from Roche Diagnostics GmbH. (Mannheim, Germany). The DNA Clean & Concentrator™ kit was purchased from Zymo Research (Freiburg, Germany). Phenol:Cholorform pH 6.7/8.0 was purchased from Amresco(Solon, OH, USA). Anti β-catenin polyclonal rabbit antibody was purchased from Cell Signaling Technology Inc. (Danvers, MA, USA). Anti-Wnt3 rabbit polyclonal antibody, anti-plasminogen rabbit polyclonal antibody, β-actin horseradish peroxidase (HRP) conjugated mouse monoclonal IgG_1_ antibody, and HRP conjugated anti-rabbit goat polyclonal antibody were purchased from Santa Cruz Biotechnology Inc. (Dallas, TX, USA). Alexa Fluor^®^ 488 conjugated anti-rabbit IgG goat polyclonal IgG and rhodamine phalloidin were purchased from Molecular probes^®^ (Eugene, OR, USA).

### 4.2. Preparation of Fibrin Gel

Fibrinogen was dissolved in distilled water at a concentration of 20 mg/mL. The thrombin solution was prepared composed of 4 Unit/mL of thrombin, 5 mM of calcium chloride, and 500 μg/mL of 6-aminocaproic acid. Each of the fibrinogen and thrombin solutions was sterilized using 0.2 μm sterilizing filter. The sterilized solutions were mixed gently with a ratio of 5:1, and 2 mL of the mixture were immediately added to 60 mm TCD and incubated at 37 °C.

### 4.3. Cell Culture

Murine cementoblast cells (OCCM-30) were maintained in Dulbecco’s Modified Eagle’s Medium (DMEM) supplemented with 10% (*v*/*v*) fetal bovine serum (FBS) and 100 units/mL penicillin/streptomycin (P/S). For the experiments, the cells were seeded onto 60 mm TCD and a fibrin gel described above. After one day of seeding, the cultured media was changed to differentiation media (DM; 10% FBS, 100 units/mL PS, 10 mM Glycerol-2-phosphate, and 50 µg/mL *L*-ascorbic acid). There were 5.25 × 10^5^ cells per 60-mm tissue culture dish which was exactly same as our previously published study [1]. All of the above cells were incubated at 37 °C under 5% of CO_2_.

### 4.4. Reverse Transcription PCR and Quantitative Real-Time PCR

Total RNA was isolated using RNA extraction reagent (RNAiso Plus) from cells cultures for three days in DM on TCD or Fibrin gel. The concentration of RNA was determined with the Nanodrop™ 2000 (Thermo Fisher Scientific, Inc., Waltham, MA, USA). The cDNA was synthesized from the total RNA with a reverse transcription kit (PrimeScripts™ RT reagent kit). Quantitative polymer chain reaction (qPCR) was performed on 7500 Real-Time PCR System (Applied Biosystems™, Carlsbad, CA, USA) with the SYBR^®^ Premix Ex Taq™ kit according to the manufacturer’s instructions. Each mRNA expression was normalized against glyceraldehyde-3-phosphate dehydrogenase (GAPDH) mRNA expression. The primer sequences are showed in Table S1. 

### 4.5. Western Blot Analysis

Cell lysates were prepared with a lysis buffer consisting of 0.1% sodium dodecyl sulfate (SDS), 0.5% sodium deoxycholate, 30 μL/mL aprotinin, 10 mg/mL phenylmethylsulfonyl fluoride, 100 mM, and sodium orthovanadate in phosphate buffered saline (PBS). The samples were subjected to 10% SDS-polyacrylamide gel electrophoresis (PAGE) acrylamide gels and then transferred onto a polyvinylidene difluoride membrane. The membrane was blocked with 5% nonfat dried milk in tris buffered saline with 0.1% tween 20 (TBS-T) for 1 h at room temperature, and incubated with each primary antibody overnight at 4 °C. This procedure was followed by incubation with an HRP-conjugated secondary antibody for 2 h. After treatment with an enhanced chemiluminescence reagent (WEST-ZOL™) treatment, the luminescence was detected with a Fujifilm LAS-2000 (Fuji, Tokyo, Japan).

### 4.6. Immunofluorescence Assay

To determine β-catenin translocation to the nucleus, OCCM-30 cells were seeded onto fibrin and TCD in growth medium. After overnight culturing, the medium was changed to a differentiation medium and cultured for three more days. A 20 mM LiCl treatment was done for the last 24 h as a control group. The cultured cells were fixed with 4% formaldehyde in PBS, for 15 min. For permeabilization, the fixed cells were incubated in PBS containing 0.1% Triton X-100 (PBS-T), for 10 min. Then, the cells were blocked with 5% BSA in PBS-T for 1 h. Samples were then incubated with a 1:200 dilution of anti-β-catenin rabbit polyclonal antibody for 2 h followed by a 1:100 dilution of Alexa Fluor^®^ 488 conjugated-goat anti-rabbit secondary antibody for 1 h. To visualize the actin filaments, the samples were incubated with rhodamine phalloidin for 20 min. Cover slips were mounted in a mounting medium contained DAPI, to identify the nuclei. The signal was detected by immunofluorescence microscopy (OLYMPUS FV300). For all of the above procedures, after each incubation step, the cells were washed for 5 min × 3 times with PBS.

### 4.7. β-Catenin/Tcf Luciferase Assay

Fibrin gel was prepared for 24 well plates using 300 μL per well. OCCM-30 cells were seeded onto the fibrin and TCD (well without fibrin gel) at a density of 1 × 10^5^ cells per well. After one day, the medium was changed to the differentiation medium. On the second day of differentiation, the cells were transfected with TopFlash or FopFlash constructs with the Genefectin™ reagent. *Renilla* luciferase constructs were used as a control. After 6 h of transfection, the medium was changed to a fresh differentiation medium without antibiotics. After 36 h of transfection, 300 µL of Dual-Glo^®^ Luciferase reagent were added to the wells containing 300 µL of medium and mixed for 10 min to lyse the cells completely. Then, 150 µL of each lysate were dispensed into a well on a 96-well assay plate and measured for firefly luminescence with the GloMax^®^-Multi Detection System from Promega Co. (Madison, WI, USA). The *Renilla* luminescence was recorded after addition of 75 µL of the Ducal-Glo^®^ Stop & Glo^®^ reagent following the manufacturer’s instructions. The TOP/FOP activity was calculated by the ratio of firefly: *Renilla* luminescence. The experiment was repeated two times independently.

### 4.8. Knockdown Assays with siRNA

The siRNAs against Wnt3a and β-catenin were used to knockdown expressions of Wnt3a and β-catenin. A scrambled siRNA (siGENOME Non-Targeting siRNA #1) was used as a control. After OCCM-30 cells were seeded on fibrin and in 60 mm TCD and incubated overnight, the growth medium was changed to the differentiation medium. One day later, each specific siRNA was transfected into the cells following the manufacturer’s instruction. After 48 h of transfection, the cells were harvested and the expression of proteins and DNA were determined.

### 4.9. DNA Constructs and Site-Directed Mutagenesis

The plasminogen promoter regions D-900 (bp-900 to +54) and D-100 (bp-100 to +54) were cloned in to the pGL3 vector following standard protocols. The primers used for the cloning of the plasminogen promoter are described in Table S2. Mutation of the LEF1 binding sites was carried out by site-directed mutagenesis. Mutant plasmids M-292 and M-282 were produced from D-900 while M-53 was produced from the D-100 plasmid. Oligonucleotides designated for the generation of mutants are listed in Table S3.

### 4.10. Transient Transfection Assay

For the transient transfection studies, plasminogen promoter constructs of D-900, D-100, mutants (M-292, M282, and M-53) or an empty basic plasmid pGL-3 as a control were co-transfected with the pRL-TK vector using the Genefectine™ reagent into the OCCM-30 cells cultured on fibrin and TCD. Six hours after the transfection, the cultured medium was changed to the differentiation medium without any antibiotics and cultured for an additionally 36 h prior to harvesting. The luciferase activity was measured with the Dual-Glo^®^ Luciferase reagent as described above in the *β-catenin/Tcf luciferase assay* section.

### 4.11. Chromatin Immunoprecipitation (ChIP)

The ChIP assay was performed according to the instructions provided by the Protein G Agarose/Salmon Sperm DNA kit. After three days of differentiation, cells were fixed by adding 37% formaldehyde stock directly into the media in cultured dishes with a final concentration of 1%, incubated for 10 min at room temperature, and quenched with 0.125 M glycine. The cell were collected by scraping and washed with cold PBS. Swelling buffer (25 mM Hepes, 1.5 mM MgCl_2_, 10 mM KCl, and 0.1% NP-40, pH 7.8) with protease inhibitor was added to the cells and incubated for 20 min on ice. Under the cold condition, the nuclei were separated with the Dounce Homogenizer (ten strokes on ice) and spun down (4000 g, 5 min). The nuclei lysis buffer contained 1% SDS, 10 mM EDTA, and 50 mM Tris-Cl (pH 8.0), and protease inhibitors were added to the collected nuclei pallets. The lysate was sonicated for 4 s 12 times under an ice-cold condition, to break the DNA into fragments. Then, 1 μL (1%) of each sample was saved as the input fraction. The remaining samples were diluted 10-fold with the ChIP dilution buffer (1% Triton X-100, 150 mM NaCl, 2 mM EDTA pH 8.0, and 20 mM Tris pH 8.0) containing a protease inhibitor. Immunoprecipitation was performed by adding protein G beads (1:10) and anti β-catenin rabbit polyclonal antibody (1:50) or protein G beads only as the negative control and incubated on a rotating shaker for 1 h at 4 °C. The bead-samples were washed three times with 0.1% SDS in dilution buffer, and one time with final washing buffer (1% Triton X-100, 500 mM NaCl, 2 mM EDTA pH 8.0, and 20 mM Tris pH 8.0) containing a protease inhibitor and two times more with TE buffer (10 mM Tris-Cl and 1 mM EDTA, pH 8.0). The cross-link was reversed by incubation in TE buffer containing 1% SDS at 65 °C for 5 h. Then, proteinase K was added (final 0.4 mg/mL) and incubated at 45 °C for 1 h. After that, the DNA fragments were extracted with Phenol/Chloroform/Isoamyl methods, and purified with DNA Clean & Concentrator™. The purified DNA was amplified by PCR using the primers which are listed in Table S4. 

### 4.12. Statistical Analysis

The PASW Statistics 17.0 (SPSS Inc., Chicago, IL, USA) was used to statistically analyze data which were presented using means ± standard deviation (S.D.). All data were analyzed with the Student *t*-test using a two-tailed test with homogeneity of variances for the data. The *n*-value for the individual test was three, but the number of repeated experiments was two or three for the statistical reliability and reproducible agreement in this study. Differences were considered to be significant if the *p* value < 0.05.

## 5. Conclusions

Fibrin positively affected OCCM30 differentiation through the canonical Wnt signaling pathway; however, canonical Wnt signaling also influenced the enzymatic degradation of fibrin. Wnt3a expression and the sequential canonical Wnt signaling were increased, and plasminogen expression was also upregulated. Therefore, fibrin has a critical limitation in supporting mineralized tissue neogenesis and possibly leads to attachment loss and apoptosis, simultaneously. Although our previous study used aminocaproic acid to temporally inhibit rapid fibrinolysis, these findings provide critical insights that spatiotemporal and precise controls of fibrinolysis are significantly required for cementoblast differentiation and tissue formation at optimal time periods.

## Supplementary Materials

Supplementary materials can be found at www.mdpi.com/1422-0067/18/11/2380/s1.

## Abbreviations

ECM	Extracellular matrices
PDL	Periodontal Ligament
t-pA	tissue-type plasminogen activator
u-pA	Urokinase-type plasminogen activator
PAI-1	Plasminogen activator inhibitor-1
α2Ap	α2-antiplasmin
TOP	TCF optimal motif
FOP	Far from optimal motif
TCD	Tissue culture dish
PCR	Polymerase chain reaction
siRNA	Small interfering ribonucleic acid
TCF	T-cell factor
LEF	Lymphoid enhancer factor
Runx2	Runx-related gene 2
ALP	Alkaline phosphatase
BSP	Bone sialoprotein
OCN	Osteocalcin
MMPs	Matrix metalloproteinases
DAPI	4′,6-diamidino-2-phenylindole
EDTA	Ethylenediaminetetraacetic acid
SDS	Sodium dodecyl sulfate
PAGE	Polyacrylamide gel electrophoresis
