# Supplementary material for: Fibrin-Enhanced Canonical Wnt Signaling Directs Plasminogen Expression in Cementoblasts
Source: Int J Mol Sci. 2017 Nov 9;18(11):2380. doi: 10.3390/ijms18112380 (PMC5713349; doi:10.3390/ijms18112380)
Supplement: Supplementary file 1 [file ijms-18-02380-s001.pdf]

Table S1. Sequences of primers used in qPCR

Gene (Mouse)	Primer Seq. (5'→3')		Access No. (Gene Bank)
Alp	Forward	GGGGACATGCAGTATGAATT	NM_007431.3
	Reverse	GGCCTGGTAGTTGTTGTGAG	
Bsp	Forward	GAGACGGCGATAGTTCC	NM_008318.3
	Reverse	AGTGCCGCTAACTCAA	
Ocn	Forward	TGAACAGACTCCGGCG	NM_007541.3
	Reverse	GATACCATAGATGCGTTTG	
Osterix	Forward	CGGGTCAGGTACAGTG	NM_130458.4
	Reverse	ACCATGACGACAAGGG	
Runx2	Forward	CTTCATTCGCCTCACAAAC	NM_001146038.2
	Reverse	GTCACTGCGCTGAAGA	
Plasminogen	Forward	GCTGCCTGTGATTGAGAACA	NM_008877.3
	Reverse	CCGTGAGACACGAACGTAGA	
Wnt3a	Forward	CATGCACCTCAAGTGCAAATG	NM_009522.2
	Reverse	TGAGGAAATCCCCGATGGT	
Mmp2	Forward	CACACCAACACTGGGACCTG	NM_008610.3
	Reverse	AGAATGTGGCCACCAGCAAG	
Mmp13	Forward	GCTTAGAGGTGACTGGCAAACT	NM_008607.2
	Reverse	TCTGGTGAAATTCAGTGGTGTC	
Mmp14	Forward	GCAAGGCTGATTTGGCAACC	NM_008608.4
	Reverse	TGGCATACTCGCCCACCTTA	
Mmp16	Forward	CTGACAAGATCCCTCCACCTAC	NM_019724.4
	Reverse	GTGTTGAAGTTCCCATCACAGA	
Mme	Forward	TCCGCTGTACAGACACTGTTTT	NM_001289463.1
	Reverse	TAGGTTGCATAGAGAGCGATCA	
Gapdh	Forward	AGGTCGGTGTGAACGGATTTG	NM_001289463.1
	Reverse	TGTAGACCATGTAGTTGAGGTCA	

Table S2. Sequences of primers for the cloning of plasminogen promoter

promoter region	**Primer Seq. (5'→3')**	
D-900	Forward	TAACGCGTCACACATGTGTGTGTGTGTGT
	Reverse	TACTCGAGACTGGCCAACAGCACCTGGAC
D-100	Forward	TAACGCGTCTGTTCGAGGTAATGTTTGCT
	Reverse	TACTCGAGACTGGCCAACAGCACCTGGAC

Table S3. Oligonucleotides designated for the generation of mutants

mutant label	**Oligonucleotides Seq. (5'→3'), mutated bases in lowercase**	
L1	Forward	TGGGAGGCACTCAGtcAgtGAAGAAGAGAGAAAGAAATGAGAGGAGAC
	Reverse	GTCTCCTCTCATTTCTTTCTCTCTTCTTCacTgaCTGAGTGCCTCCCA
L2	Forward	TGGGAGGCACTCAGtcAgtGAAGAcGctAGAAAGAAATGAGAGGAGAC
	Reverse	GTCTCCTCTCATTTCTTTCTagCgTCTTCacTgaCTGAGTGCCTCCCA
L3	Forward	GCATACAGTGGTGGGGGCCCtcGcGAATAATTAACCTATTTGGACT
	Reverse	AGTCCAAATAGGTTAATTATTCgCgaGGGCCCCCACCACTGTATGC

Table S4. Sequences of primers used in PCR after ChIP

label	**Primer Seq. (5'→3')**	
Region A	Forward	CTTGGCAGAATCTGGCATATGG
	Reverse	TACACCCTGTTACACCTCTCCG
Region B	Forward	GCCATCACTTCCAGCATCTACCAC
	Revers	CATCCACAAGCAAGGTAGTCCA

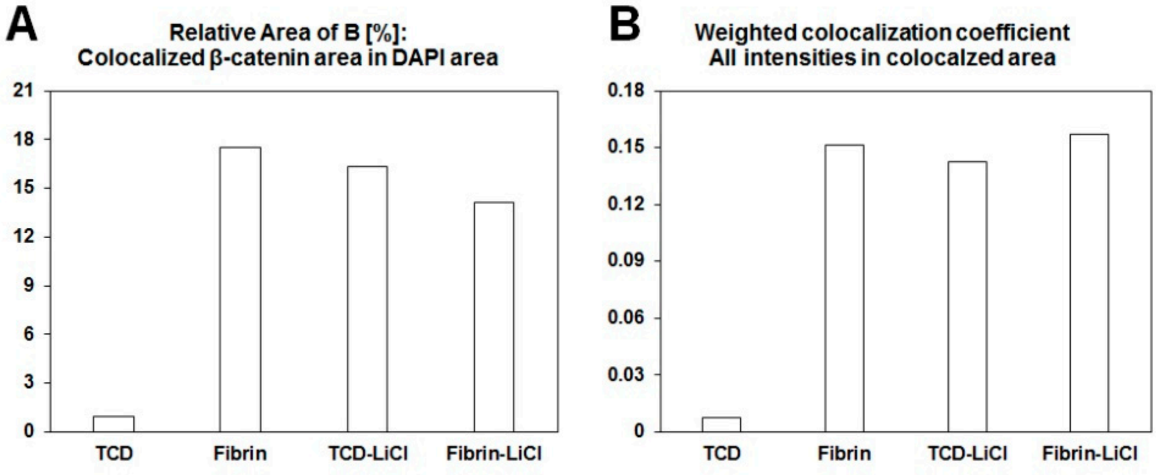

$$\text{Relative Area of B [\%]} = \frac{\text{Colocalization } \beta\text{-catenin area}}{\text{DAPI-stained nucleus}}$$

$$\text{Weighted colocalization coefficient} = \frac{\Sigma \text{ Intensities of colocalized } \beta\text{-catenin}}{\Sigma \text{ Intensities of } \beta\text{-catenin}}$$

Figure S1. Compared with TCD group, fibrin groups showed the more translocation of β-catenin to nucleus. LiCl-treatments were positive controls for the forced activation of the canonical Wnt signaling.
